# Relative Importance of Human Resource Practices on Affective Commitment and Turnover Intention in South Korea and United States

**DOI:** 10.3389/fpsyg.2018.00669

**Published:** 2018-05-17

**Authors:** Jaeyoon Lee, Young Woo Sohn, Minhee Kim, Seungwoo Kwon, In-Jo Park

**Affiliations:** ^1^Department of Psychology, Yonsei University, Seoul, South Korea; ^2^Korea Counseling Graduate University, Seoul, South Korea; ^3^Korea University Business School, Korea University, Seoul, South Korea; ^4^Department of Psychology, Henan University, Kaifeng, China

**Keywords:** perceived HR practices, nation, affective commitment, turnover intention, positive affect, training, internal mobility

## Abstract

The main purpose of this study was to investigate the impact of perceived HR practices on affective commitment and turnover intention. This study explored which HR practices were relatively more important in predicting affective commitment and turnover intention. A total of 302 employees from the United States and 317 from South Korea completed the same questionnaires for assessing the aforementioned relationships. The results illustrated that among perceived HR practices, internal mobility had the most significant association with turnover intention in both the United States and South Korea. While internal mobility was a stronger predictor of affective commitment for the United States sample, training was the most important variable for predicting affective commitment in South Korea. The second purpose of the study was to examine whether individuals’ positive affect influences the relationship between perceived HR practices and affective commitment and turnover intention. In the United States, positive affect moderated the relationship between perceived HR practices and affective commitment and turnover intention such that the relationships were stronger for individuals reporting high positive affect relative to those reporting low positive affect. However, these relationships were not significant in South Korea. We discuss the implications of these results, study limitations, and practical suggestions for future research.

## Introduction

In today’s organizational climate, human resources practices (HR practices) are gaining increased attention for companies to gain competitive advantages in the global marketplace. HR practices typically include processes such as detailed recruitment and selection, training, security, and evaluation (Pfeffer, 2005). These practices play a significant role in employees’ attitudes toward their workplace (Chang et al., 2011; Abdullah and Boyle, 2015). Prior studies have assessed the role of HR practices on several outcomes, including psychological contracts (e.g., Suazo et al., 2009) and job performance (Wright et al., 2005). However, limited studies have examined the relative importance of HR practices on job attitudes and investigated whether certain HR practices are more important than others.

In order to explore the role of HR practices, the present study focused on job attitudes as reflected in affective commitment. Job attitudes are defined as a series of evaluation of one’s own workplace that establishes own attachment toward that work place (Judge and Kammeyer-Mueller, 2012). Among many variables which assess job attitudes, this study focuses on affective commitment since it measures employee’s emotional attachment which fits the goal of this study. In order to compare not only the positive outcomes between the United States and South Korea but also the negative outcomes between two countries, turnover intention is included for another outcome variable. Since job attitudes such as commitment are strongly associated with turnover (Cohen, 1993; Cohen and Hudecek, 1993; Felfe et al., 2008), the present study utilized these outcomes simultaneously.

Rhoades and Eisenberger (2002) stated that providing training and appraising performance helps employees feel supported by their company, which leads to more workplace commitment. In other words, quality HR practices can increase employees’ affective commitment toward their companies. HR practices are also associated employees’ cognition such as turnover intention. Huselid (1995) pointed out that high investment in HR practices is linked to lower turnover intention and greater employee productivity. Boselie et al. (2005) meta-analysis revealed 27 articles related to HR practices and turnover intention from 1994 to 2003. This suggests that more and more studies are realizing the importance of relationships between HR practices and job attitudes. Yet, limited studies scrutinized HR practices, and examined whether individual HR practices influenced job attitude equally or each of them has the different impacts.

Jones and Wright (1992) also suggest that HR practices are important for improving employees’ knowledge skills while enhancing motivation and courage. Such characteristics can be distinguished from other individual factors, including dispositional affect. Few studies (Cropanzano and Wright, 2001) have focused on the relationship between positive affect and job performance using experimental or longitudinal studies. Rather, several prior studies (e.g., Staw et al., 1986; Levin and Stokes, 1989) have focused more on the effects of negative affect on job-related outcomes. Even though negative affect is an important factor to consider, the present study placed an emphasis on the moderating effect of positive affect, given that positive affect may be a more critical factor underlying organizational interactions. For instance, Watson et al. (1992) claim that positive affect has a stronger effect on socially related processes; therefore, we feel that positive affect is more crucial for influencing the relationship between HR practices and job attitudes.

The main goal of the present study was to explore specific variables related to perceived HR practices that would be more or less influential for predicting both affective commitment and turnover intention in the United States and South Korea. Finally, we investigated whether individual characteristics, such as positive affect, moderate the relationship between perceived HR practices and job attitudes across both countries.

## Theoretical Background

### Relationships Between HR Practices, Nation, Affective Commitment, and Turnover Intention

Among Allen and Meyer’s (1990) three-component model of organizational commitment, this study focuses affective commitment for several reasons. Allen and Meyer defined affective commitment within an organization as employees’ desire to belong to their company and a willingness to align personal goals with company goals. Huselid (1995) demonstrated that companies invest in HR practices to develop employees’ skills; in return, companies expect employees to get motivated and feel attached to their companies. When employees are guided by progressive HR practices, it is easier for them to become more committed to their companies and understand their role behavior. Cohen (2003) also claimed that if HR practices are successfully implemented, employees develop an emotional bond with companies. Among job attitudes, affective commitment matches the most with this theory. Additional studies have also indicated that HR practices, such as training, internal promotion, and job security are closely related to affective commitment (Appelbaum et al., 2000).

Turnover intention is employees’ cognition that companies focus upon, as it directly influences financial loss. Prior studies indicate that companies investing heavily in HR practices bear higher financial losses resulting from turnover (Shaw et al., 2005). Boselie et al.’s (2005) meta-analysis demonstrated that between 1994 and 2003, one of the most popular indicators considered by researchers associated with HR practices were turnover, absenteeism, and quit rates. Accordingly, companies need to pay closer attention to the relationship between HR practices and turnover, given that appropriate HR practices should help decrease turnover rates. Furthermore, Batt (2002) observed that HR practices that included training and employment security positively impacted employee commitment and decreased turnover intention. This paper focuses on turnover intention to balance the condition for both countries. According to research from the U.S. Bureau of Labor Statistics, the median tenure with current employers was 4.6 years (Forbes, 2016); on the other hand, for South Korea, it was almost 7 years (Hsu et al., 2014). Therefore, it would not be suitable to compare turnover behavior for two countries. Even though turnover intention and turnover behavior do not have the same concepts, numerous studies (e.g., Porter et al., 1974; Low et al., 2001) revealed that turnover intention is being investigated as an appropriate factor to predict actual turnover.

### Relative Importance of HR Practice Variables on Affective Commitment and Turnover Intention

Prior studies have indicated that HR practices play an important role in affective commitment and turnover intention; however, not all HR practice variables will be equally important. The specific components that are more or less important have yet to be explored. Training provides current and new employees with skills and behaviors that are necessary for adequate job performance (Aziz and Ahmad, 2011). Previous study (e.g., Barrett and O’Connell, 2001) indicated that HR practices (i.e., training) bolstered employees’ sense of self-worth, which led to an increase in affective commitment. Rogg et al. (2001) conducted a study with franchise dealerships, revealing that training had a more significant association (*r* = 0.22) with employee commitment than policy (*r* = 0.10), and job description (*r* = 0.17). Bartlett (2001) also observed that hours spent training was positively and significantly correlated with affective commitment (*r* = 0.15), as was the case with access to training (*r* = 0.44).

Internal mobility allows employees to relocate within an organization; the most common example would be promotion to a higher rank (Dencker, 2009). Eisenberger et al. (1986) argue that once employees are promoted, employees feel a sense of appreciation from their company; as a result, there is a significant increase in loyalty. Furthermore, internal mobility tends to motivate employees to set personal goals in line with the company’s goals (Kossek and Block, 2000; Chang, 2005). Employees’ expectations regarding the benefits they could achieve increases through internal mobility and long-term employment security policies; this, in turn, increases commitment to the company (Appelbaum et al., 2000).

Also, Kwon and Rupp (2013) found that training and development (which were treated as training and internal mobility) were negatively correlated with turnover among low performing employees. This association was greater than any other HR practice relationship, including incentive compensation and selection. Furthermore, Batt and Colvin (2011) observed that internal mobility opportunities were significantly and negatively correlated with turnover, while monitoring and performance-enhancing policies were not significantly associated with turnover.

A worldwide investigation conducted by Watson Wyatt Worldwide indicated that 48% of employees quit their jobs due a lack of promotion opportunities (Lin, 2006). Foong-ming (2008) also revealed that career development opportunities and internal promotion were negatively and significantly related to turnover intention (*r* = -0.29 and -0.23, respectively). Therefore, highly developed internal mobility is crucial for companies in terms of preserving their employees. By providing employees with more flexibility, long-term relationships between the company and its employees are easier to build, which helps alleviate turnover intentions. Comparing across several HR practices, Haines et al. (2010) revealed that internal mobility was negatively correlated with turnover (*r* = -0.05) within Canadian private sector employment. Internal mobility also referred to as lateral movement or relocations in companies. However, it is much difficult for South Korean employees to have such an opportunity compared to United States employees; therefore, this study focuses on upward movement (i.e., promotion) to balance the condition between two countries.

Since several studies have illustrated that internal mobility and training are important predictors of affective commitment and turnover intention, we examined whether these variables are relatively more important than other HR practice constructs within each sample.

Hypothesis 1: Among several HR practices, training will be relatively more important positive predictor of affective commitment compared to other HR practices in both countries.

Hypothesis 2: Among several HR practices, internal mobility will be relatively more important negative predictor of turnover intention compared to other HR practices in both countries.

### Relationships Between HR Practices, Positive Affect, Affective Commitment, and Turnover Intention

Ulrich (1998) suggested that how people behave, how they feel, and what they know reflects their job attitudes. Specifically, these aspects might be influenced by an individual’s affective state. Watson and Clark (1984) defined affect as a broad range of feelings that can influence a wide range of behaviors. For instance, individuals high in positive affect are considered to be joyful and excited; individuals low in positive affect are characterized as lethargic and apathetic (Watson and Clark, 1984). Thus, individual differences in positive affect could moderate an individual’s job attitude.

By experiencing more pleasant events, individuals high in positive affect engage more in social activities and feel happier at work (Beiser, 1974; Folkman and Lazarus, 1985). These traits are also associated with affective commitment. Meyer and Allen (1987) argue that employees are more affectively committed to their workplace if they feel comfortable and competent. Therefore, positive affect may strengthen the effects of HR practices on affective commitment.

Prior studies (Watson and Clark, 1984; Tellegen, 1985) claim that positive affective traits are associated with specific personality characteristics. For instance, extraverts are known to be more socially engaged and acclimate more quickly to group settings; as a result, extraverts are less likely to leave their current company (Maertz and Campion, 2004). Furthermore, Maertz and Griffeth (2004) revealed that employees who are more satisfied with their workplace show a lower tendency toward turnover intention. Furthermore, there is overlap between positive affective traits and turnover intention traits. Thus, the effect of HR practices on turnover intention might be significantly influenced by positive affect.

Taken together, it may be the case that positive affect strengthens the facilitative effect of HR practices on affective commitment and turnover intention. Hence, individual’s personality trait such that individuals with high positive affect may demonstrate high affective commitment and low turnover intention when working for a company that possess highly developed HR practices.

Hypothesis 3: Positive affect will moderate the positive relationship between perceived HR practices and affective commitment, such that this relationship will be stronger for individuals high (relative to low) in positive affect.

Hypothesis 4: Positive affect will moderate the negative relationship between perceived HR practices and turnover intention, such that this relationship will be stronger for individuals high (relative to low) in positive affect.

## Materials and Methods

### Sample and Procedures

For the South Korean sample, 317 respondents were selected for final analyses. One hundred and eighty-one (59%) were men, and 130 (41%) were women. The average age was 35.91 (*SD* = 7.13), and the average years of job experience was 9.68 (*SD* = 6.42). 81% of the sample was working an office job. For those in an office job, entry level/staff positions comprised the largest proportion (35.3%), followed by assistant manager (23%) and manager (21.5%).

For the South Korean employees, we requested an online survey from an agency (“Invite”). Participants were provided a web-link to an online survey. “Invite” randomly distributed the survey to employees across 467 respondents. We only included participants who had a full-time job and were over the age of 19. Furthermore, we thought that a certain amount of work experience was necessary for evaluating a company’s HR practices; thus, we removed respondents who had less than 1 year of experience. For the present study, we emphasized participants’ perception toward their current companies’ HR practices; as a result, we did not include participants who had multiple years of experience from other companies but had less than 1 year of experience in the company for which they answer the questionnaire. Also, in order for HR practices to be fully operationally, we determined that companies should have at least 100 employees. Finally, we wanted employees working for companies that had significant exposure to, and were influenced by, various HR practices; therefore, we restricted participants’ job titles to staff, assistant manager, manager, and supervisor. To evaluate nation as a whole, instead of focusing on few companies, we decided to include as many companies as possible. Also, it is crucial to avoid bias toward certain companies. As a result, for the final analyses, we assigned one company per participant; therefore, if multiple employees from one company answered the survey, we randomly selected one employee for that company, and did not include rest of the employees. As a results, we did not include 150 companies for the aforementioned reasons and other factors such as not providing a name or having more than one respondents.

For the United States sample, 302 respondents were selected for the final analysis. One hundred forty-six (48.3%) were men, and 156 (51.7%) were women. The average age was 35.90 (*SD* = 10.18), and the average years of job experience was 11.41 years (*SD* = 9.27). 48% were working office jobs. Among those in an office job, most were in entry level/staff positions (39.4%), followed by managers (33.1%) and assistant managers (24.2%).

Participants were selected through Amazon’s Mechanical Turk marketplace (MTurk). Participants were provided a web-link to an online survey. A total of 431 employees initially responded; however, 129 respondents were not suitable for the final analyses. Employees from 10 companies were not included as they did not finish the survey. Nine participants were either students or housewives and had to be removed. Also, we removed respondents from 4 companies who had less than 1 year of experience. Based on this criterion, respondents from 60 companies were removed from the analyses because their companies had less than 100 employees. Moreover, respondents from 3 companies with higher job positions were not included in our analyses. 43 companies were removed due to the duplication of companies.

This study was conducted in accordance with the Declaration of Helsinki and was approved by Korea University’s review board. All participants gave written informed consent in accordance with the Declaration of Helsinki.

### Measures

To measure HR practices, training and job description were based on Rogg et al. (2001) measures. Also, internal mobility, employment security, results-oriented appraisals, and incentive reward were created from Sun et al. (2007). These variables were selected since they illustrated both a resource-based and a control-based approach to the measurement of HR practices (Bamberger and Meshoulam, 2000). For employees in South Korea, we translated the English version of the survey into Korean and back-translated for validation. Criterion variables and moderating variables were also initially translated to Korean version and performed back translation for validation. At the end of the survey, we asked participants’ nationality to certify the appropriateness of the study. For nation, we created a dummy variable with “0” for South Korea and “1” for the United States.

### Predictors

#### Training

Five items evaluated the efficiency in how training programs are developed and distributed to employees. Example items are: “Training programs are consistently evaluated,” and “Training plans are developed and monitored for all employees.” All items were rated on a 5-point Likert-type scale, ranging from (1) strongly disagree to (5) strongly agree. Cronbach’s alphas for the United States and Korean versions were both 0.89.

#### Internal Mobility

Two questions evaluated whether employees have a future with their company or have the potential for promotion. Example items are: “Employees who desire a promotion have more than one potential position to which they could be promoted,” and “Employees have clear career paths in this organization.” Cronbach’s alpha for the United States version was 0.67 and 0.66 for the Korean version.

#### Employment Security

Two questions asked whether employees felt safe in their companies. These items included “Employees in this job can be expected to stay with this organization for as long as they wish” and “Job security is almost guaranteed to employees in this job.” Cronbach’s alpha was 0.70 for the United States version and 0.73 for the Korean version.

#### Results-Oriented Appraisals

Three questions were used to measure results-oriented appraisals. These questions evaluated whether the company appraises employees with objective indicators. Example items are “Performance appraisals are based on objective quantifiable results” and “Performance is more often measured with objective quantifiable results.” Cronbach’s alpha for the United States version was 0.80; Cronbach’s alpha for the Korean version was 0.85.

#### Incentive Reward

Two questions were used to assess incentive reward. These questions evaluated the link between job performance and reward. These items included “Bonuses are close tie or matching of pay to individual/group performance” and “Individuals in this job receive bonuses based on organizational profits.” Cronbach’s alpha for the United States version was 0.62; Cronbach’s alpha for the Korean version was 0.75.

#### Job Description

Two questions were used to measure job description. These questions evaluated whether companies possess formal written job descriptions, available to employees. These items were rated on a 6-point scale, ranging from (1) 0% to (6) 100%. An example item is “Percentage of employees who have accurate job descriptions are …” Cronbach’s alpha for the United States version was 0.60; Cronbach’s alpha for the Korean version was 0.84.

#### Composite Variable

For analyzing the moderating effect of positive affect, we created a composite variable which contains all of the HR variables we measured to represent HR practice. Since these variables were measured using different scales, we used unit-weighted z scores to combine the variables (Ackerman and Cianciolo, 2000; Song et al., 2013). Bamberger and Meshoulam (2000) demonstrated that in order to properly measure HR practices, it is crucial to containing both a resource-based and a control-based HR strategies. Therefore, they categorized HR practices into three subsystems which are people flow, appraisal and reward, and employment relation which incorporate both approaches. Each of the HR practices measured in this study is reflected in these subsystems; as a result, we standardized each variables using SPSS and added those terms to create a composite variable called ‘HR practice.’

#### Control Variables

To test for moderating effects, several variables were considered as control variables, including age and gender. Age was preferred to job tenure for the present study since few participants did not indicate the time in the organization. Previous studies (e.g., Hunt and Saul, 1975; Bedeian et al., 1992) indicated that both age and job tenure affected job attitudes; therefore, the present study chose age as a control variable. For gender, we created a dummy variable (0 = “male”, 1 = “female”).

### Criterion Variables

#### Affective Commitment

Seven items from Meyer and Allen (1984) were used to measure affective commitment. These questions asked participants whether they felt emotionally attached to their company or just consider their work as “a job.” Example items are: “I would be very happy to spend the rest of my career with this organization,” and “This organization has a great deal of personal meaning to me.” All items were rated on a 7-point Likert-type scale, ranging from (1) strongly disagree to (7) strongly agree. Cronbach’s alpha for the United States version was 0.92; Cronbach’s alpha for the Korean version was 0.76.

#### Turnover Intention

Three items created by O’Reilly et al. (1991) were used to assess turnover intention. These questions ask participants to report their desire to quit or move to another company. Example items are “I have seriously thought about leaving this company” and “If I have my way, I won’t be working for this company a year from now.” All items were rated on a 7-point Likert-type scale, ranging from (1) strongly disagree to (7) strongly agree. Cronbach’s alpha for the United States version was 0.85; Cronbach’s alpha for the Korean version was 0.80.

### Moderating Variables

#### Positive Affect

Ten items from Watson et al. (1988) Positive and Negative Affect Schedule (PANAS) were used to measure positive affect. Participants indicated the extent to which they typically felt a variety of specific emotions (e.g., “Interested,” “Excited,” and “Proud”) on a 5-point Likert-type scale, ranging from (1) very slightly or not at all to (5) extremely. Cronbach’s alpha for the United States version was 0.93; Cronbach’s alpha for the Korean version was 0.83.

### Analyses

In order to assess the moderating effect of the positive affect, we used hierarchical regression analyses (Cohen et al., 1983). To predict the outcome variables, we first entered age and gender as control variables in Step 1. In Step 2, we entered HR practices, positive affect, and the interaction term. The interaction term was created by multiplying mean-centered HR practice and positive affect variables in order to alleviate multicollinearity effects (Jaccard and Turrisi, 2003). After checking for the moderation effect, we produced an interaction plot to determine the direction of the interaction based on Cohen and Cohen’s procedure.

Several studies have used traditional multiple regression analyses to assess the relative importance of each predictor on a dependent variable. However, multiple regression analyses can be affected by multicollinearity issues. Therefore, standard multiple regression cannot properly weight the importance of predictors when multicollinearity is present (Johnson, 2000). In this case, a multiple regression may overestimate the most important predictor or underestimate the least important predictor (Johnson, 2000). Multicollinearity issues are also applied to structural equation modeling as well (Grewal et al., 2004). Johnson’s relative weights analysis alleviates this problem by creating new sets of predictors, which are orthogonal to the original predictor. Since HR practices are related to each other, it would be appropriate to use this method for analyzing the relative importance of HR variables. Therefore, this method would provide a more accurate result when figuring out which predictor is relatively more important than other predictors on criterion variables (Tonidandel and LeBreton, 2011). As a result, to examine true relative weights, the present study also adopted Johnson’s relative weights procedure (Johnson, 2001). Tonidandel and LeBreton (2014) provide a website, which automatically performs a relative weights analysis. This site provides a significance test based on bootstrapping with 10,000 replications and 95% confidence intervals, which is recommended by Tonidandel et al. (2009). We performed the procedure four times, once per criterion variable, turnover intention, and affective commitment, for both the United States and South Korean samples. The procedure reveals the raw weights for each predictor which represents relative effect sizes (LeBreton et al., 2007). Comparing these weights within the sample will illustrate which HR practices are relative more important than other HR practices on each criterion variables for each country. We will explore whether these results are identical for both countries. We also included results from the traditional multiple regression analyses to assess the direction of the relationships between HR practices, affective commitment, and turnover intention.

## Results

### Descriptive Statistics and Correlations

**Table 1** presents a correlation matrix containing means, standard deviations, and correlations between each variable. For the United States sample, all of the perceived HR practices were positively correlated with affective commitment and negatively correlated with turnover intention (all *p*s < 0.05). For the South Korean sample, except for written policy, all of the HR practices were positively correlated with affective commitment; on the other hand, most of the HR practices were negatively correlated with turnover intention (all *p*s < 0.05). Incentive reward was not significantly related to turnover intention.

**Table 1:** Descriptive Statistics and Inter Correlation

Variables	*M*	*SD*	1	2	3	4	5	6	7	8	9	10	11	12
**United States**														
(1) Age	35.90	10.18	–											
(2) Gender	1.52	0.50	0.04	–										
(3) HR Practice	0.00	0.67	–0.12*	–0.02	–									
(4) Training	3.39	0.91	–0.04	–0.08	0.74**	–								
(5) Inter Mobility	3.26	0.74	–0.03	–0.06	0.70**	0.53**	–							
(6) EmploySecurity	3.61	0.93	–0.13*	0.04	0.59**	0.33**	0.33**	–						
(7) Appraisal	3.46	0.89	0.00	0.01	0.76**	0.42**	0.54**	0.36**	–					
(8) Reward	3.00	1.06	–0.15**	–0.06	0.59**	0.28**	0.31**	0.15**	0.40**	–				
(9) Description	4.37	1.11	–0.03	0.03	0.57**	0.37**	0.31**	0.19**	0.31**	0.12*	–			
(10) Positive Affect	3.47	0.89	0.04	0.14*	0.40**	0.34**	0.38**	0.22**	0.31**	0.21**	0.18**	–		
(11) Affcommitment	4.51	1.53	0.07	0.05	0.54**	0.47**	0.55**	0.35**	0.44**	0.17**	0.30**	0.53**	–	
(12) Turnover	3.65	1.81	–0.13*	–0.07	–0.45**	–0.40**	–0.50**	–0.29**	–0.34**	–0.15*	–0.25**	–0.42**	–0.74**	–
**South Korea**														
(1) Age	35.91	7.13	–											
(2) Gender	1.41	0.49	–0.24**	–										
(3) HR Practice	0.00	0.78	0.00	–0.10	–									
(4) Training	3.10	0.74	–0.00	–0.02	0.81**	–								
(5) Inter Mobility	2.88	0.46	0.02	–0.05	0.47**	0.32**	–							
(6) EmploySecurity	3.16	0.82	–0.02	–0.05	0.69**	0.48**	0.31**	–						
(7) Appraisal	3.06	0.79	–0.02	–0.09	0.86**	0.64**	0.36**	0.49**	–					
(8) Reward	3.04	0.86	0.01	–0.12*	0.75**	0.51**	0.27**	0.33**	0.63**	–				
(9) Description	3.24	0.1.20	0.10	–0.13*	0.78**	0.56**	0.34**	0.44**	0.61**	0.52**	–			
(10) Positive Affect	3.18	0.50	0.03	–0.13*	0.46**	0.31**	0.05	0.27**	0.43**	0.41**	0.38**	–		
(11) Affcommitment	4.12	0.80	0.15**	–0.08	0.45**	0.46**	0.31**	0.36**	0.38**	0.31**	0.35**	0.28**	–	
(12) Turnover	4.48	1.16	–0.23**	0.05	–0.16**	–0.17**	–0.33**	–0.16**	–0.13*	–0.08	–0.16**	0.05	–0.56**	–

*M, Mean; SD, Standard Deviation; Inter Mobility, Internal Mobility; EmploySecurity, Employment Security; Appraisal, Result-Oriented Appraisal; Reward, Incentive Reward; Description, Job Description; Turnover, Turnover intention; Affcommitment, Affective Commitment; ^∗∗^*p* < 0.01, ^∗^*p* < 0.05.*

### Relationships Between Perceived HR Subgroups and Affective Commitment and Turnover Intention

**Table 2** illustrates results from the multiple regression and relative weights analyses for both countries. Each relative weights analysis was performed for each criterion variable. The first three columns indicate beta values from the regression analysis, importance weights, and rescaled importance weights from the relative weights analysis for the United States sample; the last three columns provide these values for the South Korean sample. The first row displays effects on affective commitment, and the second rows displays effects on turnover intention. Importance weights are interpreted as the proportion of each variance’s contribution to the criterion value (Tonidandel and LeBreton, 2014); therefore, higher relative weights are related to a higher portion of a model’s*R*^2^. The sum of the total importance weights is the model’s *R*^2^ value. The rescaled importance weights are the percentage of the importance weights’ contribution to a model’s *R*^2^ value (Johnson, 2004). Therefore, a higher percentage is interpreted as a higher proportion of the *R*^2^ value. Beta values were included for comparison purposes and to assess discrepancies between beta values and importance weights.

**Table 2:** Multiple regression and relative importance of perceived HR practices on affective commitment and turnover intention in United States and South Korea.

	United States			South Korea		
	*β*	*IW*	*RIW (%)*	*β*	*IW*	*RIW (%)*
**Dependent Variable – Affective Commitment**				
Training	0.19**	0.09*	23.50	0.29**	0.09*	33.33
Internal Mobility	0.33**	0.14*	37.12	0.15**	0.04*	15.80
ES	0.12*	0.05*	12.09	0.13*	0.05*	17.30
ROA	0.14*	0.07*	17.96	0.02	0.03*	12.47
Incentive Reward	–0.07	0.01	1.71	0.04	0.02*	8.82
Job Description	0.07	0.03	7.62	0.06	0.03*	12.28
*R^2^*	0.38			0.26		
*IW*		0.38			0.26	
*RIW*			100			100
**Dependent Variable -Turnover Intention**				
Training	–0.15*	0.06*	21.75	–0.08	0.01	8.04
Internal Mobility	–0.36**	0.13*	45.53	–0.31**	0.09*	73.40
ES	–0.10	0.03*	11.33	–0.05	0.01	7.67
ROA	–0.05	0.04*	12.61	0.04	0.00	3.14
Incentive Reward	0.05	0.00	1.66	0.06	0.00	1.39
Job Description	–0.05	0.02	7.11	–0.04	0.01	6.35
*R*^2^	0.29			0.12		
*IW*		0.29			0.12	
*RIW*			100			100

*ES, Employment Security; ROA, Result-Oriented Appraisal; IW, Importance Weight; RIW, Rescaled Importance Weight. Importance weights are relative weights which sum to the *R*^2^ of the full model. Rescaled importance weight are calculated by dividing the importance weight by the *R*^2^ of the model; ^∗∗^*p* < 0.01, ^∗^*p* < 0.05.*

The first rows of **Table 2** illustrate results of perceived HR practices on affective commitment for both countries. For the United States sample, the *R*^2^ for the model was 0.38. The relative weights analysis revealed that training (*IW* = 0.09, *p* < 0.05), internal mobility (*IW* = 0.14, *p* < 0.05), employment security (*IW* = 0.05, *p* < 0.05), results-oriented appraisals (*IW* = 0.07, *p* < 0.05) were significantly predictive. **Table 2** shows that the largest proportion of explained variance in affective commitment was internal mobility (37.12%), followed by training (23.50%) and results-oriented appraisals (17.96%).

For the South Korean sample, the *R*^2^ for the model was 0.26. The relative weights analysis indicated that training (*IW* = 0.09, *p* < 0.05), internal mobility (*IW* = 0.04, *p* < 0.05), employment security (*IW* = 0.05, *p* < 0.05), results-oriented appraisals (*IW* = 0.03, *p* < 0.05), incentive reward (*IW* = 0.02, *p* < 0.05), and job description (*IW* = 0.03, *p* < 0.05) were significantly predictive. Training accounted for the largest proportion of explained variance (33.33%), followed by employment security (17.30%) and internal mobility (15.80%). These results provide partial support for Hypothesis 1.

The second set of columns in **Table 2** display results for turnover intention within both countries. For the United States sample, the *R*^2^ for the model was 0.29. The relative weights analysis revealed that training (*IW* = 0.06, *p* < 0.05), internal mobility (*IW* = 0.13, *p* < 0.05), employment security (*IW* = 0.03, *p* < 0.05), and results-oriented appraisals (*IW* = 0.04, *p* < 0.05) were negative predictors. The largest proportion of explained variance in turnover intention was accounted for by internal mobility (45.53%), followed by training (21.75%) and result oriented appraisal (12.61%).

For the South Korean sample, the *R*^2^ for the model was 0.12. Here, the relative weights analysis revealed that only internal mobility (*IW* = 0.09, *p* < 0.05) was statistically significant, explaining 73.40% of the variance in turnover intention. Taken together, these results indicate support for Hypothesis 2.

### Moderating Effect of Positive Affect on the Relationship Between Perceived HR Practices and Affective Commitment and Turnover Intention

**FIGURE 1:**
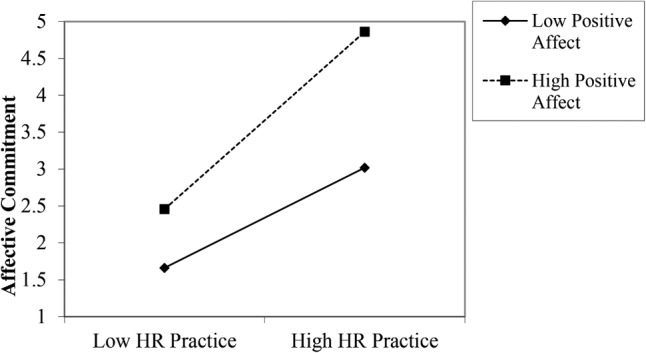
Relationship between perceived HR practices and affective commitment moderated by positive affect in United States sample.

To test Hypothesis 3, a hierarchical regression analysis was performed on the US sample to predict affective commitment. The control variables were inserted at Step 1. In Step 2, perceived HR practices, positive affect, and the interaction term were entered. This analysis revealed that 43% of the variance in affective commitment could be explained by the model (*p* < 0.05; **Table 3**). Furthermore, the interaction was a significant independent predictor (*β* = 0.11, *p* < 0.05). The interaction graph is shown in **Figure 1**. Specifically, the relationship between perceived HR practices and affective commitment was stronger for those reporting high (*β* = 0.54, *SE* = 0.22, *t* = 8.15, *p* < 0.001) relative to low positive affect (*β* = 0.35, *SE* = 0.21, *t* = 5.67, *p* < 0.001). However, this relationship was not observed in the South Korean sample (*β* = -0.01, *p* = 0.90), suggesting that support for Hypothesis 3 was only observed in the United States sample.

**Table 3:** Results of hierarchical regression analyses predicting the moderating effects of the relationships between perceived HR practices and positive affect on affective commitment and turnover intention for the United States.

	Affective Commitment		Turnover Intention	
	Model 1-1	Model 1-2	Model 2-1	Model 2-2
**Step 1**				
Age	0.07	0.12*	–0.13^∗^	–0.17^∗∗^
Gender	0.05	0.01	–0.07	–0.05
**Step 2**				
HR practices		0.41**		–0.37^∗∗^
Positive Affect		0.38**		–0.29^∗∗^
HRP × PA		0.11*		–0.14^∗∗^
*R*^2^	0.01	0.43	0.02	0.31
*Adjusted R*^2^	0.00	0.42	0.02	0.30
*F*	1.13	45.16**	3.29	26.97^∗∗^
*ΔR*^2^	0.01	0.43	0.02	0.29

*^∗^*p* < 0.05, ^∗∗^*p* < 0.01. HRP × PA: The interaction term between perceived HR practices and positive affective.*

**FIGURE 2:**
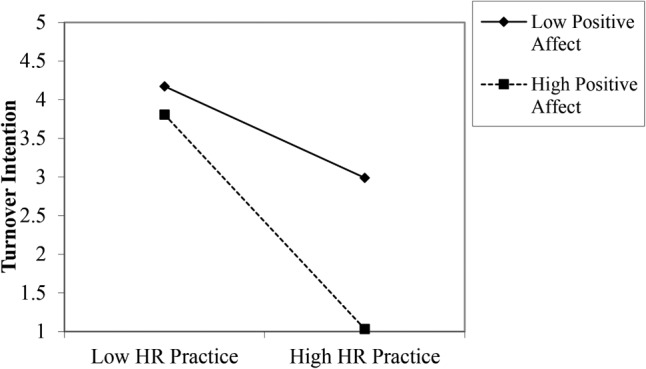
Relationship between perceived HR practices and turnover intention moderated by positive affect in United States sample.

The same procedure was conducted for turnover intention. Results indicated that 29% of the variance in turnover intention could be explained by the model (*p* < 0.01; **Table 3**). Furthermore, the interaction was a significant independent predictor of turnover intention (*β* = -0.14, *p* < 0.01). **Figure 2** displays the interaction graph. Specifically, the relationship between perceived HR practices and turnover intention was stronger among those reporting high (*β* = -0.50, *SE* = 0.29, *t* = -6.80, *p* < 0.001) relative to low positive affect (*β* = -0.29, *SE* = 0.27, *t* = -4.14, *p* < 0.001). However, this relationship was not significant in the South Korean sample (*β* = 0.08, *p* = 0.14). Thus, Hypothesis 4 was only supported within the United States sample.

## Discussion

The present study scrutinized and determined which HR practice variables were specific predictors of affective commitment and turnover intention in the United States and South Korea. Results demonstrated a consensus in terms of perceived HR practices that were relatively more predictive of both affective commitment and turnover intention in the United States. Conversely, different HR practice variables were relatively more important in predicting affective commitment and turnover intention. Finally, we observed a moderating effect of positive affect on these relationships. Specifically, positive affect was a significant moderator for the United States sample, while it did not have a significant effect on South Korean sample.

For Hypotheses 1 and 2, we investigated whether internal mobility and training were relatively more important than other HR practices on the two outcome variables. Results revealed that for the United States, internal mobility was relatively more important for both outcome variables. Conversely, for South Korea, while internal mobility was the only significant predictor of turnover intention, training was relatively more important for predicting affective commitment. Internal mobility allows employees to have more options within organizations. For instance, employees could be promoted to a higher position, which could increase commitment. As a result, employees in the United States with this line of thinking would engage in more affective commitment than if there was a focus on training. However, internal mobility could not be the most significant predictor for South Korea because of the rapid changes to the HR practice structure throughout South Korea. As South Korea’s economy has expanded, due to shifts resulting from the 1997 IMF incident, South Korean companies realized that they needed to adjust their traditional HR practices (Lee, 1998; Chang, 2002; Park and Yu, 2002; Rowley and Bae, 2002). Yet, since many HR practices were adopted from Western culture (Cox, 1994), the incongruity between HR practices and South Korean’s national value may differ the effects of HR practices. Therefore, it may be the case that well established training system within a workplace contributes the most to enhance commitment for South Korean employees. On the other hand, our results demonstrate that internal mobility was the most important predictor of turnover intention for both countries. Here, internal mobility may be considered an alternative option for turnover intention in both countries. From a practical perspective, companies perhaps could invest more in internal mobility to help with employee appeasement.

Moreover, for Hypotheses 3 and 4, we explored whether positive affect moderates the relationship between perceived HR practices and both outcome variables. Results revealed that positive affect only moderated the relationship between perceived HR practices and affective commitment and turnover intention in the United States sample. This might be explained by the nature of the aforementioned evolving HR practice systems in South Korea. Mowday et al. (1982) asserted that individuals place more effort and sacrifice for the sake of the company if they feel a strong workplace attachment. Due to progressive HR practices in the US, these relationships are rather robust. Yet, as noted by Bae and Rowley (2002), South Korea has yet to engage in highly developed HR practices; thus, individual affect may not have the same level of impact as in the United States.

### Theoretical and Practical Implications

Theoretically, our findings can address controversies surrounding the universalistic and contingency perspectives. Universalistic perspectives claim that there is a best practice for HR activities, regardless of the cultural context (Osterman, 1994; Huselid, 1995). On the other hand, contingency perspectives suggest that there are no best practices that equally affect companies; effective practices are dependent on contextual factors (Begin, 1993; Lepak and Snell, 1999). Overall, our results support both perspectives. Regarding turnover intention, both countries showed that internal mobility was relatively important, which aligns well with universalistic perspectives. However, regarding affective commitment, internal mobility was relatively significant in the United States, while training was relatively significant in South Korea. Therefore, efficacious practices were context specific in this case. Therefore, instead of trying to determine the “right” perspective, we can infer that each perspective may be important based on the job attitude being assessing.

Findings from the present study provide useful information for companies as to the type of HR practices they should implement. Even though recent articles (e.g., Cappelli, 2011) claimed that training programs are becoming less important by United States companies, our result revealed that training programs still considered as an important predictor by employees from United States. Therefore, this paper could provide useful guideline for United States companies when forming effective HR practices. Specifically, our findings support those from Björkman et al. (2007), observing that different HR practices are utilized within MNC subsidiaries across their various host countries. Therefore, if a corporation from the United States wants to establish a subsidiary in South Korea, it may be beneficial for them to invest more in training in order to increase employees’ affective commitment rather than investing in internal mobility. However, a focus on internal mobility will still be necessary for impacting turnover intention with a South Korean workforce. Furthermore, companies from South Korea may consider how individual affective states could influence their HR practices. Based on the present results, training appeared to be the most important factor for increasing affective commitment. Therefore, companies could build more advanced training programs to help employees feel more attached to their workplace. For the United States, methods for increasing positive affect could also help maintain positive attachment.

Unlike other studies, we implemented a relative importance analysis, which is a more accurate method for comparing the relative weights of certain variables on predicted outcomes. Here, we were able to eliminate multicollinearity problems typically associated with traditional multiple regression analyses (Johnson, 2000). Furthermore, even though previous studies (e.g., Weber and Tarba, 2010; Gooderham and Nordhaug, 2011) have examined HR practice efficacy in several countries, few addressed direct comparisons between countries. Thus, the present cross-cultural analysis allowed us to determine the effects of specific HR practices across countries.

### Limitations and Future Directions

A few study limitations should be noted. First, instead of distributing a survey to employees, face-to-face, we used MTurk for data collection in the United States. However, several studies (e.g., Buhrmester et al., 2011; Hauser and Schwarz, 2016) have defended MTurk’s validity in comparison with traditional, face-to-face methods; as a result, we believe the present data to be valid. Second, the self-report nature of our design could not address any behavioral aspects of these relationships. Future work should include experimental study designs to avoid any common method biases. Third, several participants did not report their job tenure, total work experiences, or the establishment of their current companies. These could be the important control variables, but the present study could not apply. More in-depth studies should consider these variables to assess HR practices’ impact.

Also, the present study did not consider other potential sources of individual differences between two countries such as power distance, individualism, or organizational culture. Therefore, the present study focuses on national difference rather than cultural difference between two countries. Even though prior research (e.g., Szirmai and Pilat, 1990; Lambert et al., 2004; Jung and Sung, 2008) contributed significant results by comparing only nation itself, further research is recommended to reflect varieties of cultural aspects for more accurate results. Lastly, the study assessed participants’ perceptions toward HR practices. As a result, instead of observing existing HR practices, we relied on employee impressions. If possible, future studies should gain access to companies’ actual HR practices to obtain more objective assessments.

Moreover, measurement invariance between two countries was not found in this study. Recently, it is emphasized by many scholars (e.g., Steenkamp and Baumgartner, 1998; van de Vijver and Leung, 2000) that it is crucial to measure whether the same construct is measured across specified cultures. Even though the present study fulfilled configural invariance, rest of the steps such as metric invariance was not achieved. Therefore, the present study did not evaluate the moderation effects of a national difference since data may not be adequate to state that the results were not affected by different response styles from each culture. As a result, this study evaluated two countries separately. Future studies could perform measurement invariance more accurately before analyzing cross-cultural data.

For the future study, additional relationships between HR practices and other criterion variables could implement the relative weights analyses employed in the present study. Prior studies, such as those illustrating the association between HR practices and line management (Sikora and Ferris, 2014), risk management (Becker and Smidt, 2016), and emotional performance (Gabriel et al., 2016), could be well served by discovering the specific HR practices that are more important predictors of job-related outcomes.

## Author Contributions

All authors listed have made a substantial, direct and intellectual contribution to the work, and approved it for publication.

## Conflict of Interest Statement

The authors declare that the research was conducted in the absence of any commercial or financial relationships that could be construed as a potential conflict of interest.
